# Targeted photodynamic therapy treatment of *in vitro* A375 metastatic melanoma cells

**DOI:** 10.18632/oncotarget.27221

**Published:** 2019-10-22

**Authors:** Channay Naidoo, Cherie Ann Kruger, Heidi Abrahamse

**Affiliations:** ^1^Laser Research Centre, University of Johannesburg, Johannesburg, South Africa

**Keywords:** metastatic melanoma, nano active targeting, zinc phthalocyanine tetra-sulphonic acid, photodynamic therapy

## Abstract

Metastatic Melanoma (MM) is a deadly form of skin cancer and many photodynamic therapy (PDT) studies have noted limitations in relation to effective photosensitizer (PS) drug uptake in tumors. The focus of this study was to develop a PS multicomponent nanoparticle drug conjugate carrier system which specifically targets MM cells via biomarkers to actively enhance PS delivery and so improve MM PDT. An antibody-metallated phthalocyanine-polyethylene glycol-gold nanoparticle drug conjugate, was successfully synthesized and characterized. PS active drug targeting PDT experiments at 673 nm were conducted within *in vitro* cultured MM. Results noted that this drug conjugate enhanced the PDT treatment of MM, through improved subcellular localization of the PS, as well as noted significantly improved cytotoxic and late apoptotic cellular death in cells. The results from this study demonstrate that through the bio-active antibody PS drug targeting of MM, the efficacy of PDT treatment for this cancer can be enhanced.

## INTRODUCTION

Melanoma represents about 10% of all skin malignancies, however due to its ability to metastasize and spread, it accounts for more than 80% of all skin cancer related deaths, with approximately 100000 fatalities annually [1, 2]. There are currently various cancer treatments available for melanoma such as chemotherapy, surgery, biological therapy and radiotherapy or immunotherapy, however, these treatments are sometimes invasive and induce harsh unwanted side effects. Therefore, investigation into unconventional forms of treatment for melanoma such as PDT administered alone or in combination with other therapies need to be conducted to reduce the side effects and be non-invasive [3]. In relation to immunotherapy the standard of care of MM patients, include immunomodulating modalities such as anti-PD-1 drugs (nivolumab, pembrolizumab) and anti-CTLA-4 antibody ipilimumab which have provided “proof of concept” for further research in the field of immunooncology, however to date this treatment option still remains unavailable [4].

PDT involves the administration of a light sensitive non-toxic drug known as a PS which passively localizes in tumor cells [3, 5] When this PS is irradiated with visible red light (620 - 690 nm), it becomes excited and so forms cytotoxic reactive oxygen species (ROS) [3, 5]. ROS accomplishes photo-cytotoxicity by promoting cellular oxidative stress, which induces cell death and so causes tumor destruction [5].

PDT is a feasible treatment modality for cancer, however, in relation to MM various studies have noted some PS drawbacks [2, 3]. Since conventional PS drugs can only passively diffuse into tumor cells, it has been noted that sometimes they are absorbed by healthy tissues, causing undesirable side effects and tend to be absorbed in lowered concentrations than originally administered [6]. Additionally, PSs are recognized as foreign matter by biological barriers and so *in vivo* tend to be destroyed by immune system barriers, resulting in a poor drug uptake [7]. Moreover, solid melanoma tumors are seemly more resistant to PDT, due to poor absorption of PS drugs and limitations of laser light being able to reach these cells [3]. Literature has noted positive PDT treatments of MM with zinc sulpho phthalocyanine (ZnPcS_mix_) PSs, due to their longer light wavelength absorption peaks above 650 nm, being able to penetrate deep seated tumors, rich in melanin, however noted that ZnPcS tends to aggregate, due to their poor water solubility and sulpho purity, so this limited their overall PDT effectivity [8].

Numerous studies have reported that gold nanoparticles (AuNPs) can be utilized as drug carriers in PDT applications to improve PS passive uptake in tumor cells, due to their abilities to avoid biological barriers, ease of functionalization, as well as abilities to promote photothermal cell death induction due to their metallated content.[9–12] Additionally, studies have noted that Melanoma Inhibitory Activity (MIA) is an antigen, which is specially overexpressed on melanoma cells only, thus making it a highly specific and sensitive biomarker for MM drug uptake targeting [13]. Thus, the conjugation of a MM tumor-targeting antibody (Ab) such as Anti-MIA, onto a sulpho pure ZnPcS PS carrying AuNPs surface would seem highly desirable, in order to promote drug solubility, as well as active MM tumor targeting uptake in order to enhance PDT seems promising [14–16]. Furthermore, studies have reported that Zinc phthalocyanine tetra-sulphonic acid (ZnPcS_4_) is more soluble than ZnPcS_mix_, due to its tetra sulphonated groups [8]. Additionally, studies have determined the threshold for acute toxicosis of parenterally administered ZnPcS_4_, in mice and evaluated the compound’s safety in a phase I clinical trial of ZnPcS_4_ based PDT in pet dogs with naturally occurring tumors. These animal studies confirmed the good tolerability and systemic safety of ZnPcS_4_, as no changes in immunological, behavioral and organic parameters could be detected upon treatment with the non-photoactivated ZnPcS_4_ and so show the extraordinary photoactive potential of the ZnPcS_4_ as a photosensitizer for PDT [17–20].

**Figure 1:**
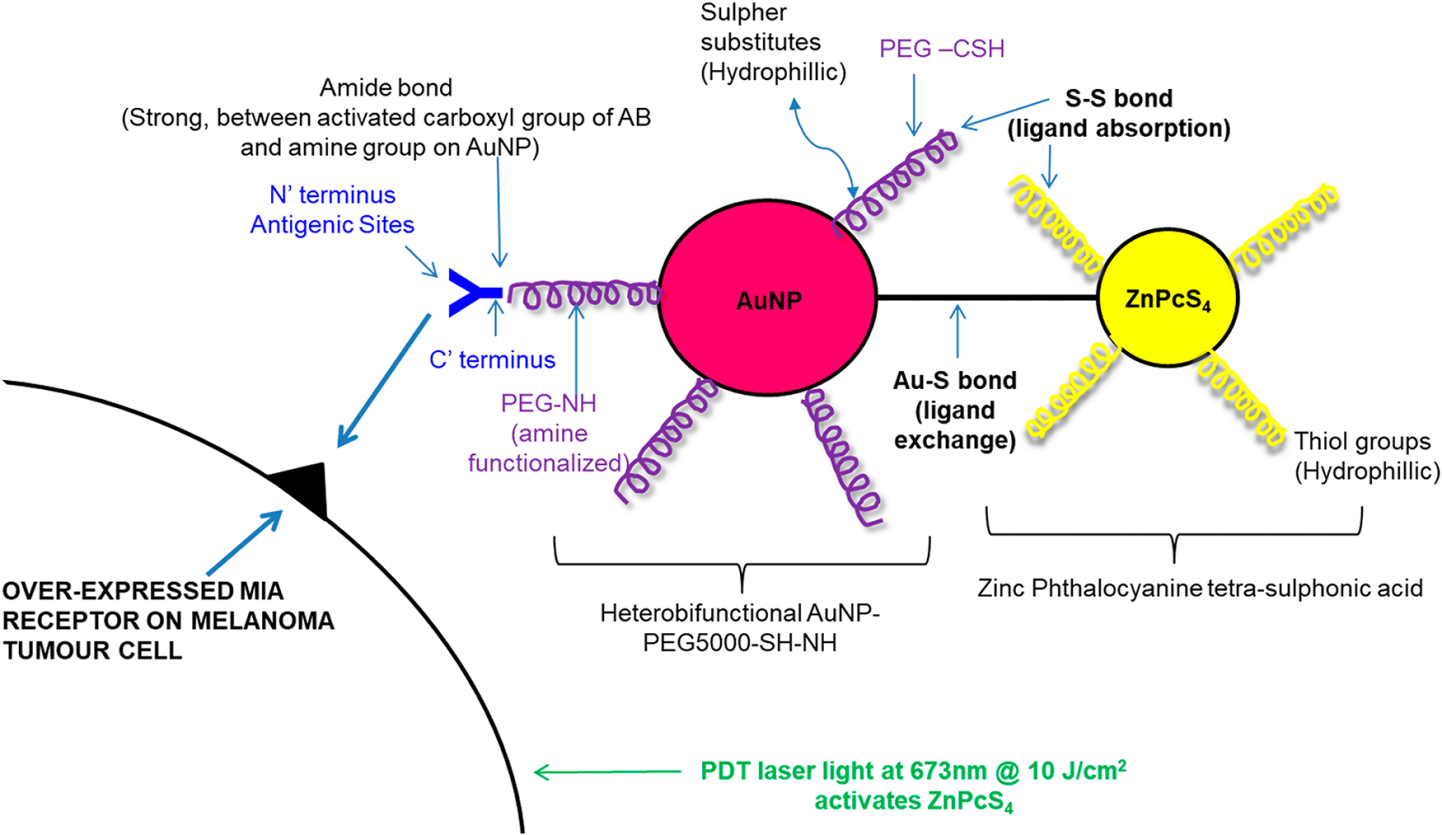
Theorized active targeted final PS molecular drug conjugate ZnPcS_4_ – AuNP-PEG5000-SH-NH_2_ – Anti-MIA Ab structure and bond formation.

Thus, this study ZnPcS_4_ PS drug was conjugated onto the surface of amine functionalized AuNPs, which had Anti-MIA antibodies bound to its surface in order to actively improve PS drug delivery and increase its uptake and absorption within MM target tumor cells. The outcome of this study clearly enhanced PDT treatment for this type of skin cancer (Figure 1) [17, 18].

## RESULTS

### Molecular characterization of the final PS drug conjugate

#### UV-Visible spectroscopy

**Figure 2:**
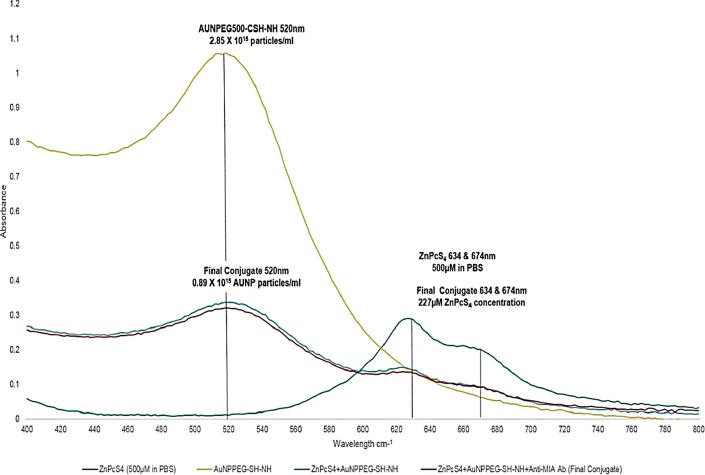
UV-Visible absorption spectra of the final molecular PS drug conjugate (ZnPcS_4_ -AuNP-PEG5000-SH-NH_2_ - Anti-MIA Ab) and various controls within the 400 to 800 nm spectral region.

**Figure 3:**
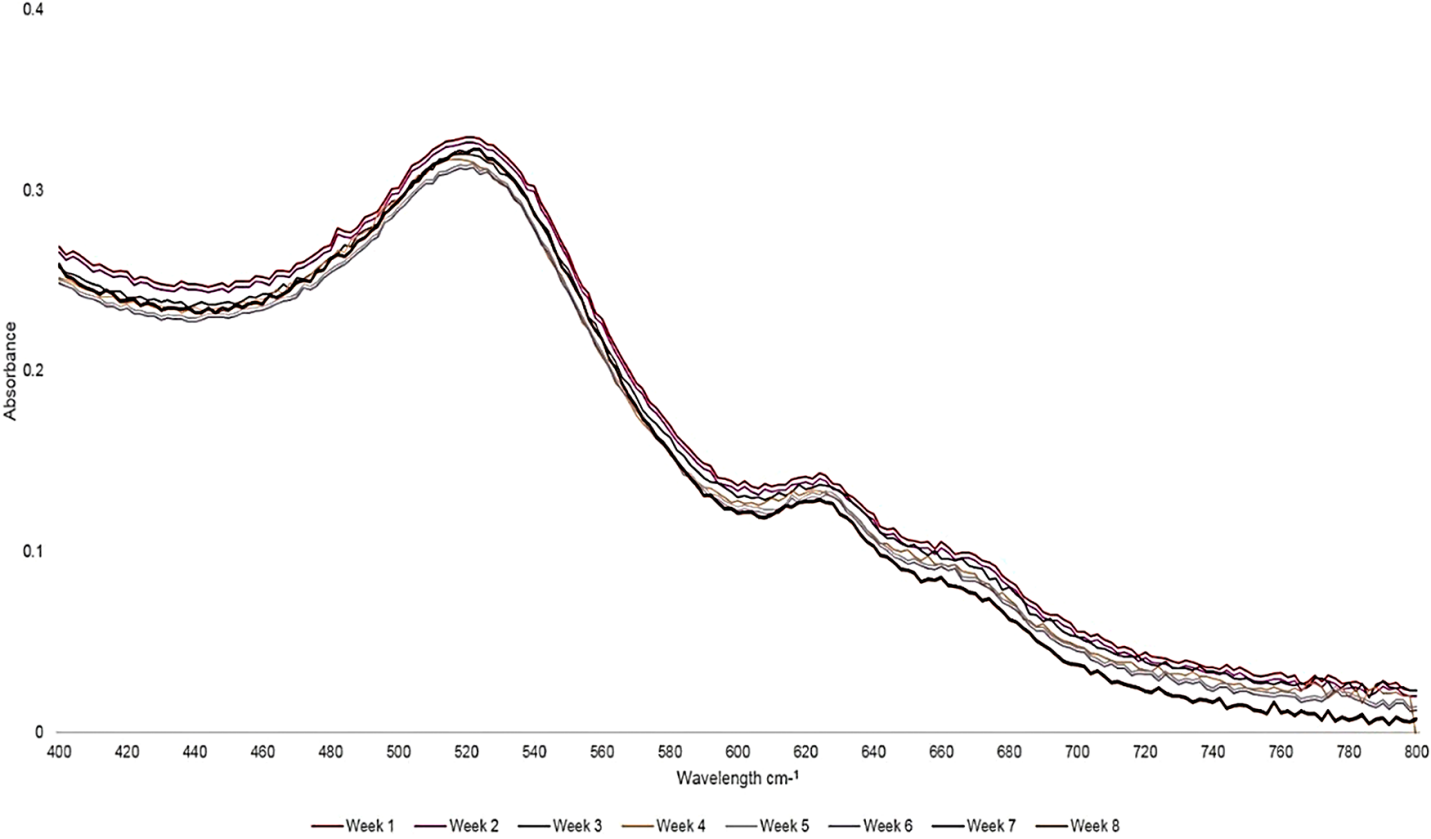
UV-Visible absorption and fluorescent photostability spectra of the final molecular PS drug conjugate (ZnPcS_4_ -AuNP-PEG5000-SH-NH_2_ - Anti-MIA Ab) recorded within the 400 to 800 nm spectral region over an 8-week period.

The absorption spectra of the final PS drug conjugate were read using the spectrum/purity scan mode within the 400-800 nm spectral region (Figure 2). 500 µM of ZnPcS_4_ noted two major Q bands of emission (634 and 674 nm) within the far-red spectral range and AuNP-PEG5000-CSH-NH_2_ noted a peak absorption band of 520 nm, which equates to 2.85 × 10^15^ particles/ml [21]. When both of these absorption spectra were compared to the final PS drug conjugate, both absorption peaks still remained prominent, however lowered in absorption, confirming that ZnPcS_4_ had been successfully bound to AuNP-PEG5000-SH-NH_2_, and the PS ROS integrity, as well as AuNP photothermal properties had remained intact. When comparing the major absorption peak fold falls of the PS drug conjugate to ZnPcS_4_ and AuNP-PEG5000-SH-NH_2_ controls, it was determined that 0.89 × 10^15^ AuNP-PEG5000-SH-NH_2_ particles/ml had been successfully bound to 227 µM of ZnPcS_4_ PS drug. Since, the major absorption peaks of the final PS drug conjugate at 520 and 673 nm in relation to ZNPcS_4_ and AuNP control peaks did not broaden much, this suggests the final PS drug conjugate had good size distribution with minimal aggregation [22]. However, since the absorption peaks of the final PS drug conjugate did slightly broaden; this is indicative of definitive bonding between all the chemical components, as there is an obvious increase in molecular size [23]. Moreover, the slight shift in the resonance peak position of AuNP at 520 nm, within the final molecular conjugate was also indicative that ZnPcS_4_ and Anti-MIA Ab had successfully bound to its surface [23]. Finally, over 8 weeks of the experimental assays the absorption spectra Q bands of 520, 634 and 674 nm stayed consistent, suggesting the final PS drug conjugate remained photostable and retained its photothermal properties after molecular synthesis and overtime (Figure 3).

**Figure 4:**
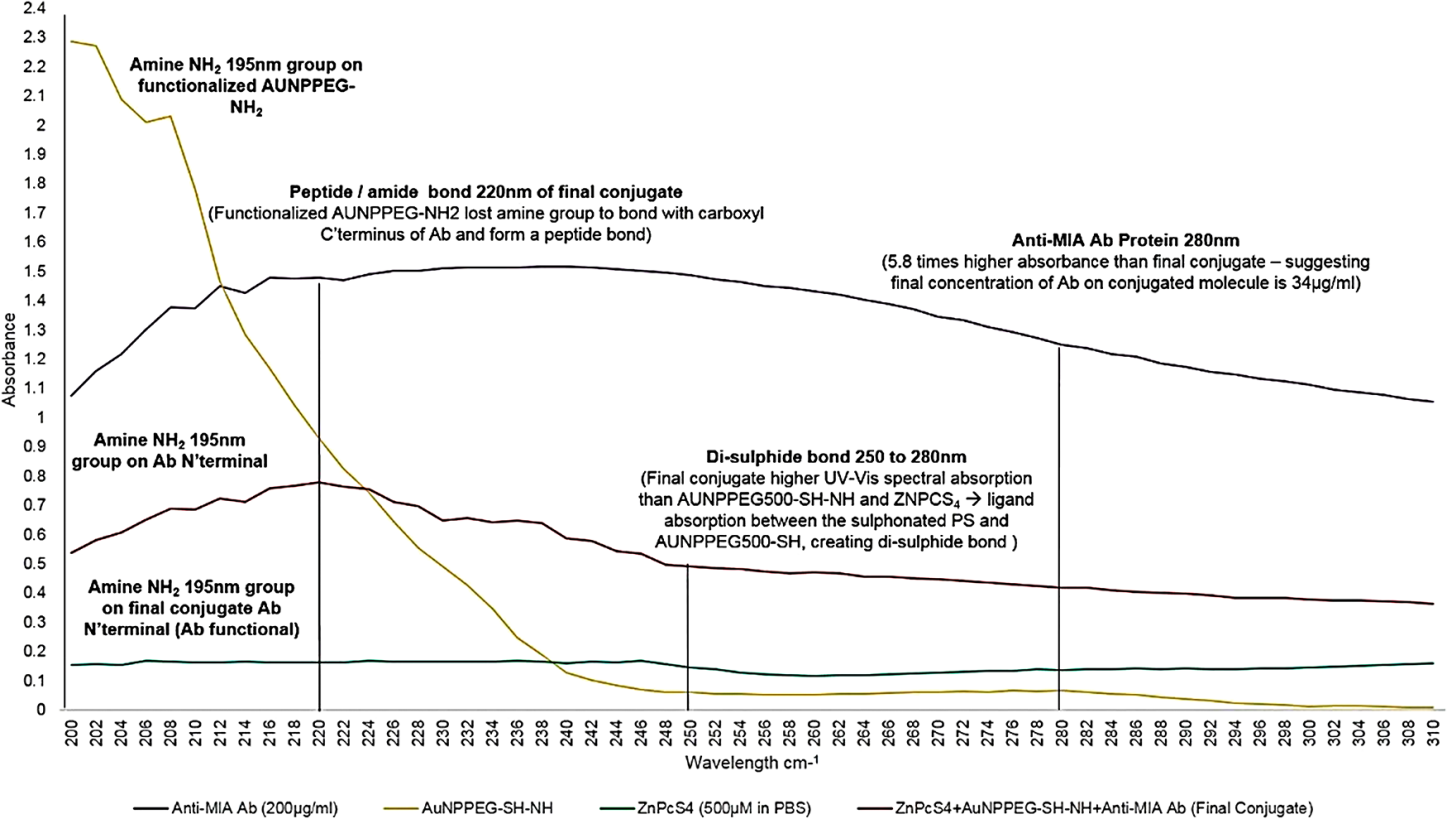
UV-Visible protein direct absorption spectra of the PS drug conjugate and various controls within the 200 to 310 nm spectral region.

With reference to Figure 4, the protein direct UV 280 nm spectral region of the final PS drug conjugate noted a 5.8 absorbance fold fall when compared to control 200 µg/ml Anti-MIA Ab within the same spectral range. This finding suggests that only 34 µg/ml of Anti-MIA Ab was successfully bound to the final molecular PS drug conjugate. According to literature, amine (NH_2_) functional groups produce a high absorption peak at a wavelength spectrum of 195 nm, whereas the condensation of an amine with a carboxylic acid to produce an amide peptide bond (CO-NH) tend to produce a high absorption peak at a wavelength spectra of 220 nm [24]. The AuNP-PEG5000-SH-NH_2_ control noted a high NH_2_ group absorption peak at a wavelength spectrum of 195 nm, which is expected since it was NH_2_ functionalized. Additionally, the Anti-MIA Ab control also noted a high NH_2_ group absorption peak at 195 nm, which is predictable considering its n’ terminal has NH_2_ functional groups. Whereas, the final PS drug conjugate noted lowered NH_2_ group absorption peak at 195 nm, when compared to that of the AuNP control, suggesting that NH_2_ group on the AuNP had condensed with the activated carboxylic group on the activated c’ terminus of the Abs to produce a CO-NH bond. The final CO-NH bond was additionally confirmed, by observing the high 220 nm absorption peak within the final PS drug conjugates spectra. Moreover, since the final PS drug conjugate still noted the presence of NH_2_ functional groups at the 195 nm absorption peak, this suggested that the n’ terminus active targeting sites of the Anti-MIA Ab within the final multicomponent active nano PS molecular drug conjugate remained unaffected, correctly orientated (i. e. bound to the AuNP-PEG5000-SH-NH_2_ at c’ terminus) and so were considered functional for active PS drug delivery. Lastly, the final PS drug conjugate had a higher absorption spectra within the 250 to 280 nm range than when compared to AuNP and ZnPcS_4_ controls, suggesting possible ligand absorption between the sulphonated ZnPcS_4_ PS and AuNP-PEG5000-SH-NH_2_, had occurred in order to create di-sulphide bond [25].

#### FT-IR spectroscopy

**Figure 5:**
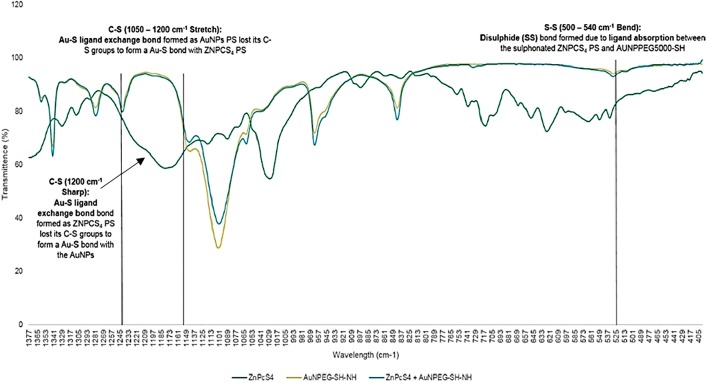
FT-IR spectra analysis for confirmatory ligand exchange (Au-S) and absorption (S-S) bond confirmation between AuNP-PEG5000-SH-NH_2_ and ZnPcS_4_ within the final PS drug conjugate.

FT-IR spectroscopy has various applications in structural identification, and can be used for qualitative analysis of molecular ligand and absorption bond formations, as well as peptide bond identification within the final PS drug conjugate (Figure 5) [26–29]. FT-IR characterization results revealed the presence of Au-S ligand exchange bonding, since the spectra of control conjugate ZnPcS_4_ - AuNP-PEG5000-SH-NH_2_, noted a C-S (1050-1200 cm^–1^) stretch shift, suggesting AuNP-PEG5000-SH-NH_2_ lost their C-S groups to bond with ZnPcS_4_ [26, 27]. Moreover, the presence of Au-S ligand exchange bonding was confirmed since the FT-IR spectra of ZnPcS_4_ - AuNP-PEG5000-SH-NH_2_ noted a loss of a C-S (1200 cm^–1^) sharp band when compared to ZnPcS_4_ alone, suggesting ZnPcS_4_ lost its C-S groups to bond with AuNP-PEG5000-SH-NH_2_ [28]. Finally, the FT-IR spectra of ZnPcS_4_ - AuNP-PEG5000-SH-NH_2_ noted a sharp S-S (500-540 cm^–1^) bend suggesting that ligand absorption between the sulphonated ZnPcS_4_ PS and AuNP-PEG5000-SH-NH_2_ occurred to form a weak di-sulphide bond [29].

**Figure 6:**
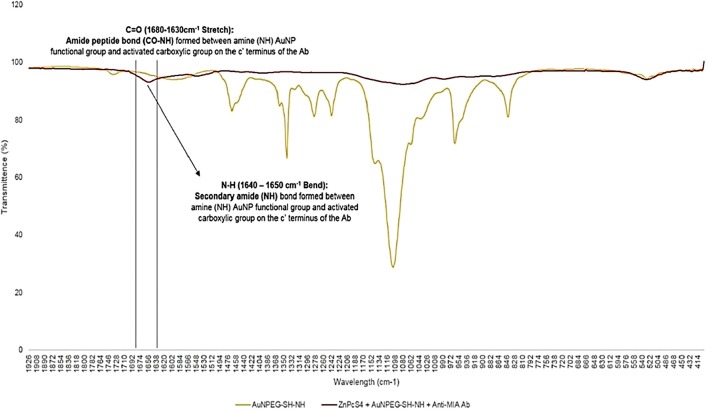
FT-IR spectra analysis for confirmatory amide (CO-NH) bond confirmation between AuNP-PEG5000-SH-NH_2_ and the activated carboxylic group on the c’ terminus of the Anti-MIA Ab within the final PS drug conjugate.

Furthermore, the final molecular conjugate (ZnPcS_4_ - AuNP-PEG5000-SH-NH_2_ - Anti-MIA Ab) was also subjected to FT-IR analysis for confirmatory amide bond analysis, by noting and identifying the formation of amide bonds when compared to the FT-IR spectra of AuNP-PEG5000-SH-NH_2_ alone (Figure 6). With referral to Figure 6, FT-IR characterization revealed that final molecular conjugate noted a C=O (1680-1630 cm^–1^) stretch and sharp N=H bend (1640-1650 cm^–1^), when compared to the AuNP-PEG5000-SH-NH_2_ alone, suggesting that strong primary and secondary amide peptide bonds (CO-NH) formed between the amine (NH_2_) functionalized group on the AuNPs and the activated c’ terminus of the Anti-MIA Ab [29].

#### Dynamic light scattering and zeta potential

**Figure 7:**
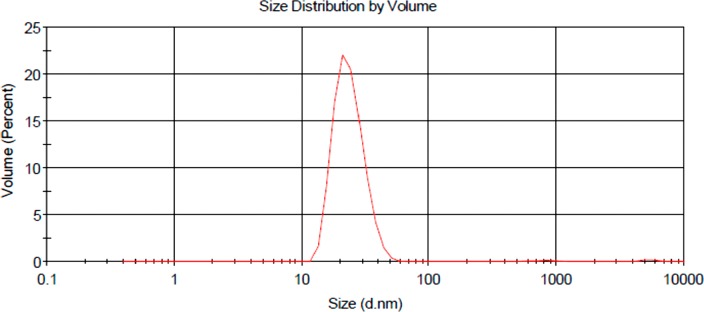
DLS hydrodynamic radius distribution graph of final molecular drug conjugate consisting of ZnPcS_4_ - AuNP-PEG5000-SH-NH_2_ - Anti-MIA Ab.

DLS and ZP results are shown in Table 1 and Figure 7. The DLS hydrodynamic radius distribution of the final PS drug conjugate noted a single narrow width peak, indicating it remained spherical with no aggregation and was homogenously pure. The final PS drug conjugate reported a mean Z-average diameter of 45.20 ± 6.458 nm, suggesting it was indeed small enough to be considered as an active nanodrug carrying system [30]. The final PS conjugate reported a PDI purity value of 0.306, signifying it was monodisperse and mostly consisted of single sized particles. The ZP value of the final PS drug conjugate was found to be 28.6 ± 3.73 mV, proposing that it was moderately stable with a slightly positively charge, hinting at the fact that it should remain stable within an *in vivo* environment, as well as be passively be taken up and retained within tumorous cells more selectively [30, 31].

**Table 1:** DLS and ZP results for characterization of final molecular NP PS drug delivery system

Sample	Mean Z-Average Diameter Measured by DLS (nm)	Polydisersity Index (PDI)	Zeta Potential (mV)
AuNP	11.06 ± 1.02	0.247 (Monodisperse)	
ZnPcS_4_	15.44 ± 2.23	0.406 (Monodisperse)	
Expected average ZnPcS_4_ - AuNP	41.94 ± 1.63		
Result average ZnPcS_4_ - AuNP	42.94 ± 3.625	0.289 (Monodisperse)	
Anti-MIA Ab	3.21 ± 1.83	0.352 (Monodisperse)	
Expected average ZnPcS_4_ - AuNP - Anti-MIA Ab	49.36 ± 2.73		
Result average ZnPcS_4_ - AuNP - Anti-MIA Ab (final molecular drug conjugate)	45.20 ± 6.458	0.306 (Monodisperse)	28.6 ± 3.73 (Moderately stable)

#### Subcellular localization

**Figure 8:**
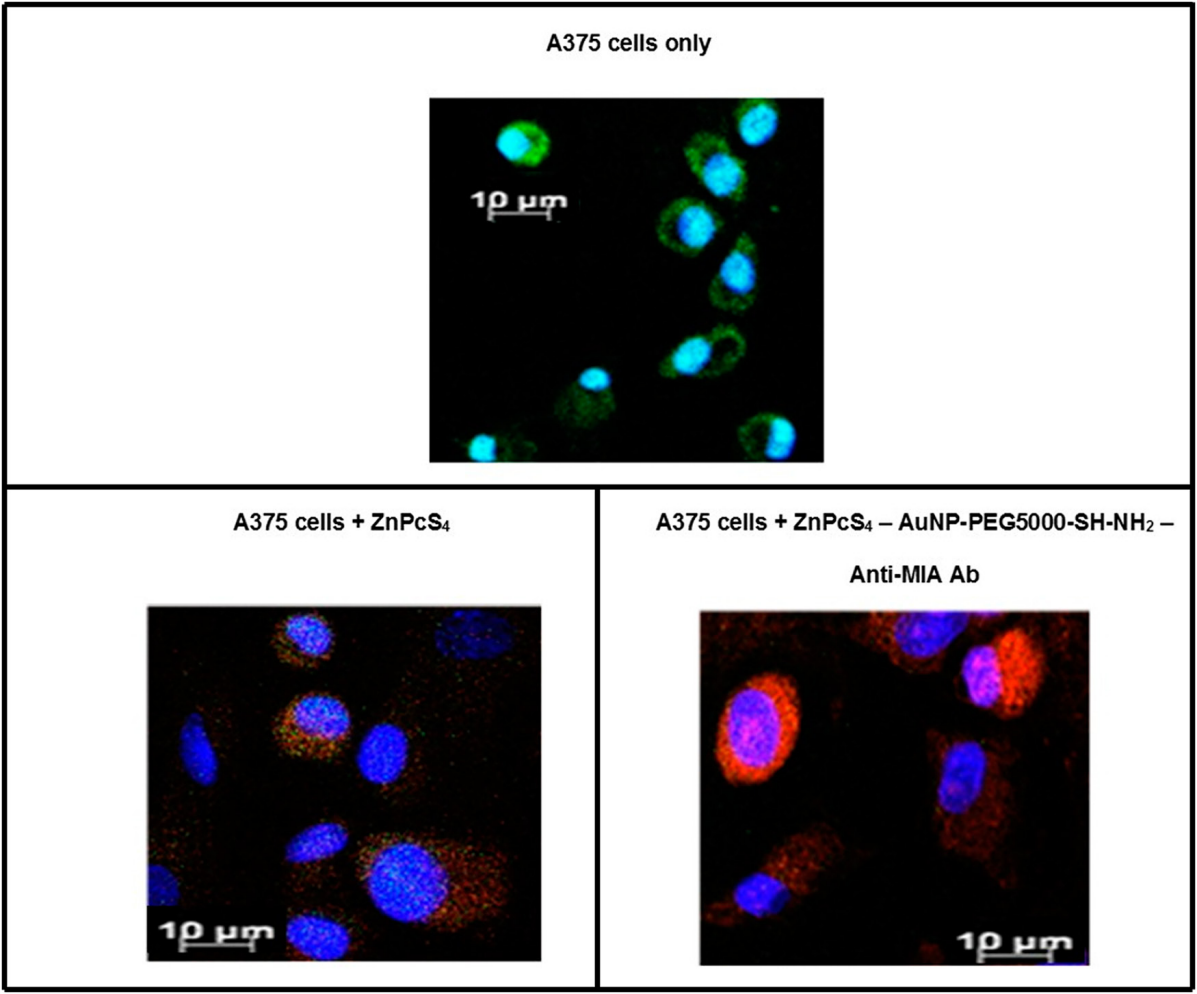
Subcellular localization results of ZnPcS_4_ PS alone verses PS drug conjugate within *in vitro* cultured MM cells. (Blue = nuclei, green = membrane proteins and red = localization of ZnPcS_4_ PS drug).

Immunofluorescent studies noted a far more predominant localization of ZnPcS_4_ PS (red) in the cytoplasm (green) and nuclei (blue) of MM cells, which received the final PS drug conjugate, than when compared control PS drug administration alone (Figure 8). These findings suggest that the targeting affinity of the PS drug conjugate in relation to Anti-MIA Ab biomarker specify for MM cells was functional, and so improved the subcellular localization and concentration uptake within these cells.

### ZnPcS_4_ PS PDT dose response assay

**Figure 9:**
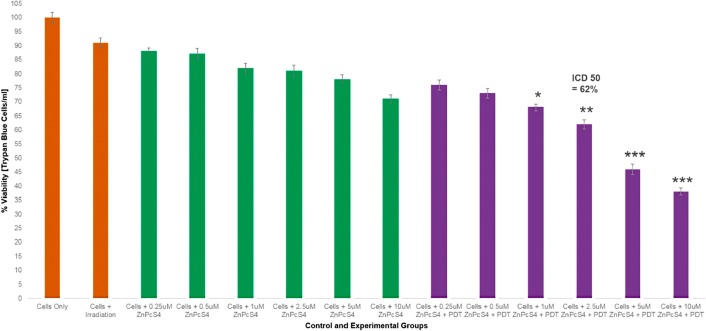
Trypan blue exclusion cell viability results of control and experimental groups of ZnPcS_4_ at various concentrations with and without laser irradiation at 673 nm at 10 J/cm^2^.

**Figure 10:**
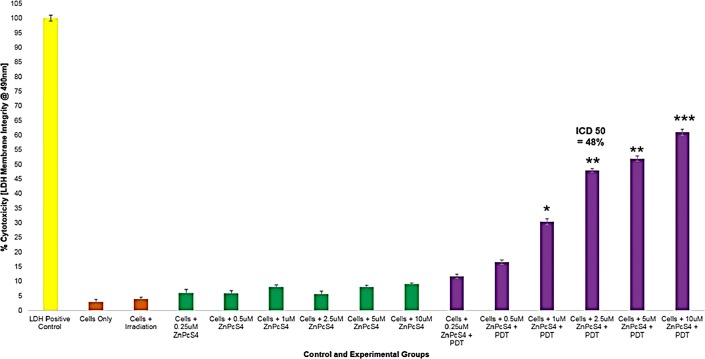
LDH cellular cytotoxicity assay results of control and experimental groups of ZnPcS_4_ at various concentrations with and without laser irradiation at 673 nm at 10 J/cm^2^.

In order to establish the IC_50_ of the ZnPcS_4_ PS, various concentrations of ZnPcS_4_ PS and/or PDT laser irradiation (at a wavelength of 673 nm using a fluency of 10 J/cm^2^) were applied to MM cells and results were reported in the form of Trypan Blue Exclusion cell viability tests (Figure 9) and Lactate Dehydrogenase (LDH) cytotoxicity and membrane integrity assay results (Figure 10).

The Trypan Blue Exclusion cell viability test (Figure 9) noted control MM cells, which received various concentrations of ZnPcS_4_, with no laser irradiation applied to them, that there was an insignificant dose dependent decrease in cell viability. This suggested that the ZnPcS_4_ in its inactivated form, when administered alone lacks dark toxicity. Additionally, control MM cells which received laser irradiation alone, also noted no cellular damage. Experimental groups of MM cells which received 0.25 and 0.5 µM of ZnPcS_4_ and laser irradiation, showed no significant decreases in cell viability. However, cells which received 1, 2.5, 5 and 10 µM of ZnPcS_4_ with laser irradiation showed dose dependent significant decreases in cell viability, when compared to the cells only control. The LDH cytotoxicity and membrane integrity assay (Figure 10) reported similar significant results within experimental groups which received 1, 2.5, 5 and 10 µM of ZnPcS_4_ with laser irradiation, while all control groups noted insignificant findings.

These results indicate that ZnPcS_4_ is an excellent PS for the use of PDT on MM, since it is capable of substantial cell death induction. However, in order to establish if the PS when administered in a drug carrying conjugate, was capable of targeted and improved PDT, the IC_50_ of 2.5 µM ZnPcS_4_ PS with laser irradiation applied was chosen, as it reported a significant decrease of 38%** in cell viability and 48%** cellular cytotoxicity. Thus, in relation to the below final conjugate studies: the concentration of the final PS drug conjugate that was administered to cells consisted of 2.5 µM ZnPcS_4_ PS, bound to 0.425 µg/ml Anti-MIA Ab with 1.11 × 10^13^ AuNP-PEG5000-SH-NH_2_ particles/ml in 0.001 M PBS.

### ZnPcS_4_ – AuNP-PEG5000-SH-NH2 – Anti-MIA ab final PS drug conjugate PDT response assays

#### Morphological assessment

**Figure 11:**
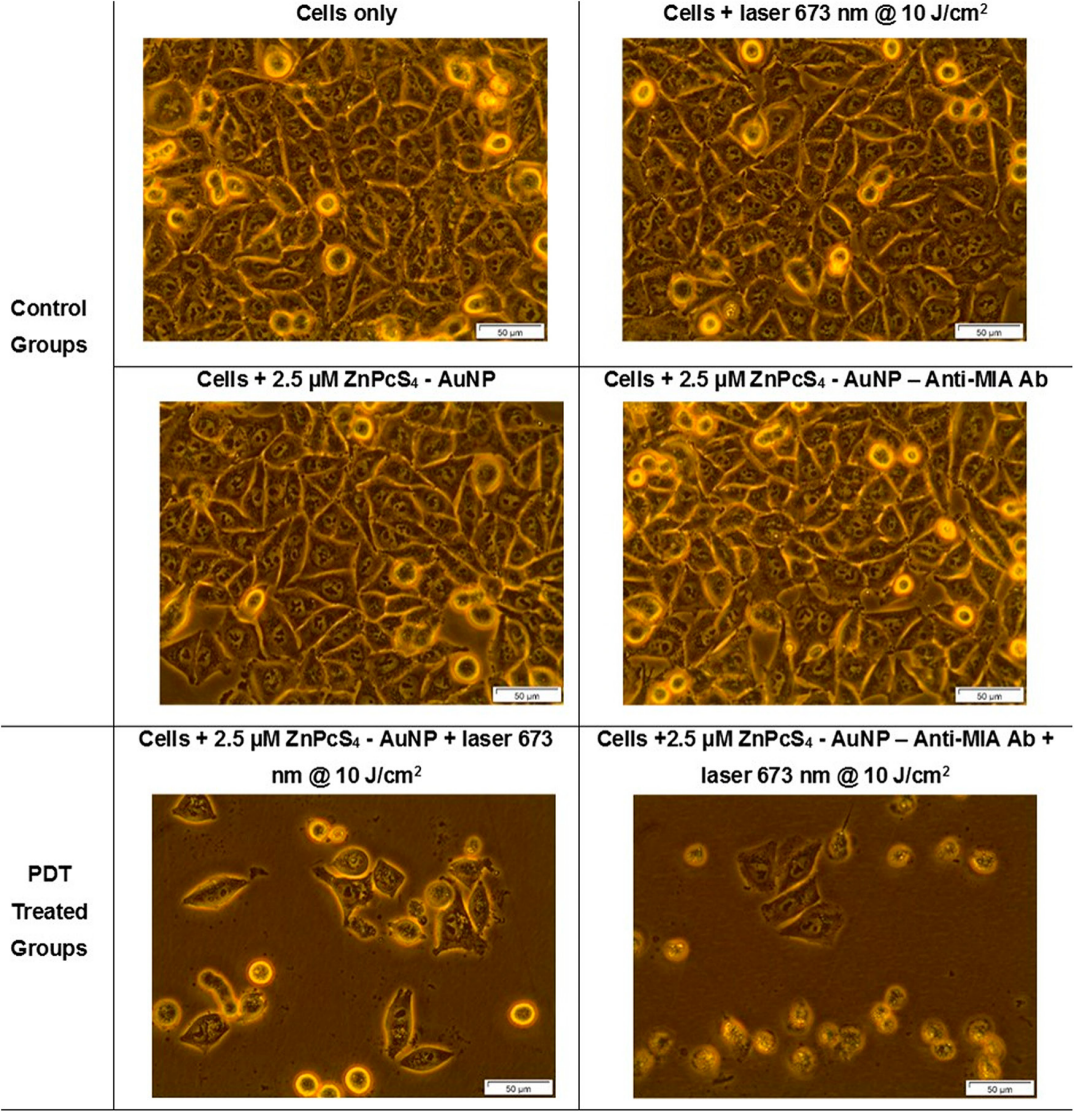
Light microscopy morphological images of MM cells at 400× magnification of control and experimental groups that were subjected to laser irradiation at a wavelength of 673 nm and a fluence of 10 J/cm^2^ during final PS drug conjugate PDT response assays.

Changes in cellular morphology of various control group and PDT experimental groups, were assessed 24 hours post irradiation by light microscopy by comparing these images to the MM cells only control (Figure 11). No significant morphological damage was noted in the cells control group which received laser treatment only, suggesting laser treatment alone doesn’t induce phototoxicity. Control cell groups which received 2.5 µM ZnPcS_4_ – AuNP-PEG5000-SH-NH_2_ or 2.5 µM final PS drug conjugate only, no significant change in cellular morphology was observed, signifying that the final molecular drug conjugate exhibited no dark toxicity.

Within the PDT treated control groups, which received with 2.5 µM ZnPcS_4_ – AuNP-PEG5000-SH-NH_2_, changes in morphological structure, such as loss of cellular shape, detachment and free-floating cells was observed. These results are supported by studies by Conde *et al.* (2012) whom noted that within PDT applications AuNPs have laser light induced photothermal properties, which assist in tumor destruction [12]. However, the most significant cellular damage was noted within the PDT treated experimental group, which received 2.5 µM of the final PS drug conjugate, as cells appeared to be completely detached, rounded up and cellular debris was seen. These findings suggest that the final PS drug conjugate Anti-MIA Ab actively enhanced cellular uptake of the ZnPcS_4_ and so enhanced the PDT treatment outcome. These findings correlate with studies by Hong *et al.* (2016), which highlight that NP PS drug uptake and absorption can be improved, when NPs are functionalized with targeting biomolecules, which allow for active and specific targeted delivery in tumor cells and so enhance PS cellular localization and overall PDT outcome [32].

#### Trypan blue exclusion test

**Figure 12:**
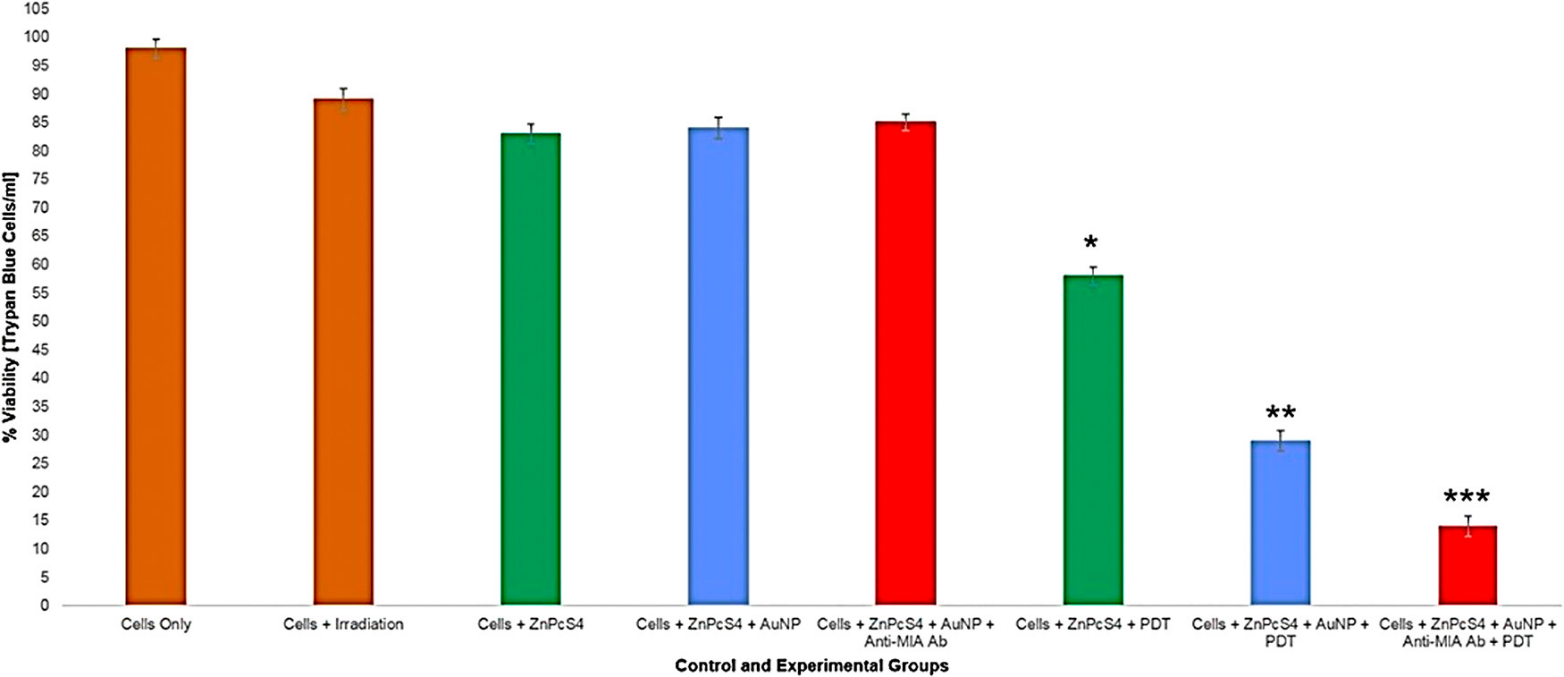
Trypan blue exclusion MM cell viability results of control and experimental groups that were subjected to final PS drug conjugate PDT response assays.

In order to determine the effect, the final PS drug conjugate had during PDT response assays, various control and experimental groups were subjected to Trypan blue exclusion staining (Figure 12). The MM cells only control group noted 98% viable cells and so this was used as reference-base line to statistically compare other results against. Control groups which received laser irradiation only, no substantial decrease in viability was found, suggesting laser irradiation alone, had no effect on cellular viability. Similarly, control cells which received 2.5 µM ZnPcS_4_ only, noted no significant decrease in cell viability, suggesting that this PS drug when administered at this concentration, lacks dark toxicity in cells. Additionally, there was no significant viability decrease observed in cell control groups which received control 2.5 µM ZnPcS_4_ – AuNP-PEG5000-SH-NH_2_ or 2.5 µM final PS drug conjugate administration alone. These findings indicate that even when the ZnPcS_4_ was bound to either AuNP-PEG5000-SH-NH_2_, as well as Anti-MIA Ab, it remained stable (in its inactive form) and so lacked dark toxicity.

The PDT treated groups, which consisted of cells with the addition of either 2.5 µM ZnPcS_4_, 2.5 µM ZnPcS_4_ – AuNP-PEG5000-SH-NH_2_ or 2.5 µM final PS drug conjugate showed varying significant decreases in cell viability. The PDT treated control group which received 2.5 µM ZnPcS_4_ with laser irradiation noted a 46%* decrease in cell viability, whereas the PDT treated control group which received 2.5 µM ZnPcS_4_ – AuNP-PEG5000-SH-NH_2_ with laser irradiation noted an even more significant decrease of 69%** in cell viability. These findings are indicative that the bound AuNPs to the PS promoted PDT photothermal induced cellular damage, than when compared to PS drug administration alone [32]. However, the most significant decrease of 84%*** in cellular viability, was noted in PDT treated experimental group which received 2.5 µM final PS drug conjugate. These findings suggest that the Anti-MIA Ab, which was conjugated to the final PS drug carrier, actively enhanced PS drug uptake in cells and so improved PDT treatment outcomes immensely.

#### LDH cellular cytotoxicity and membrane integrity assay

**Figure 13:**
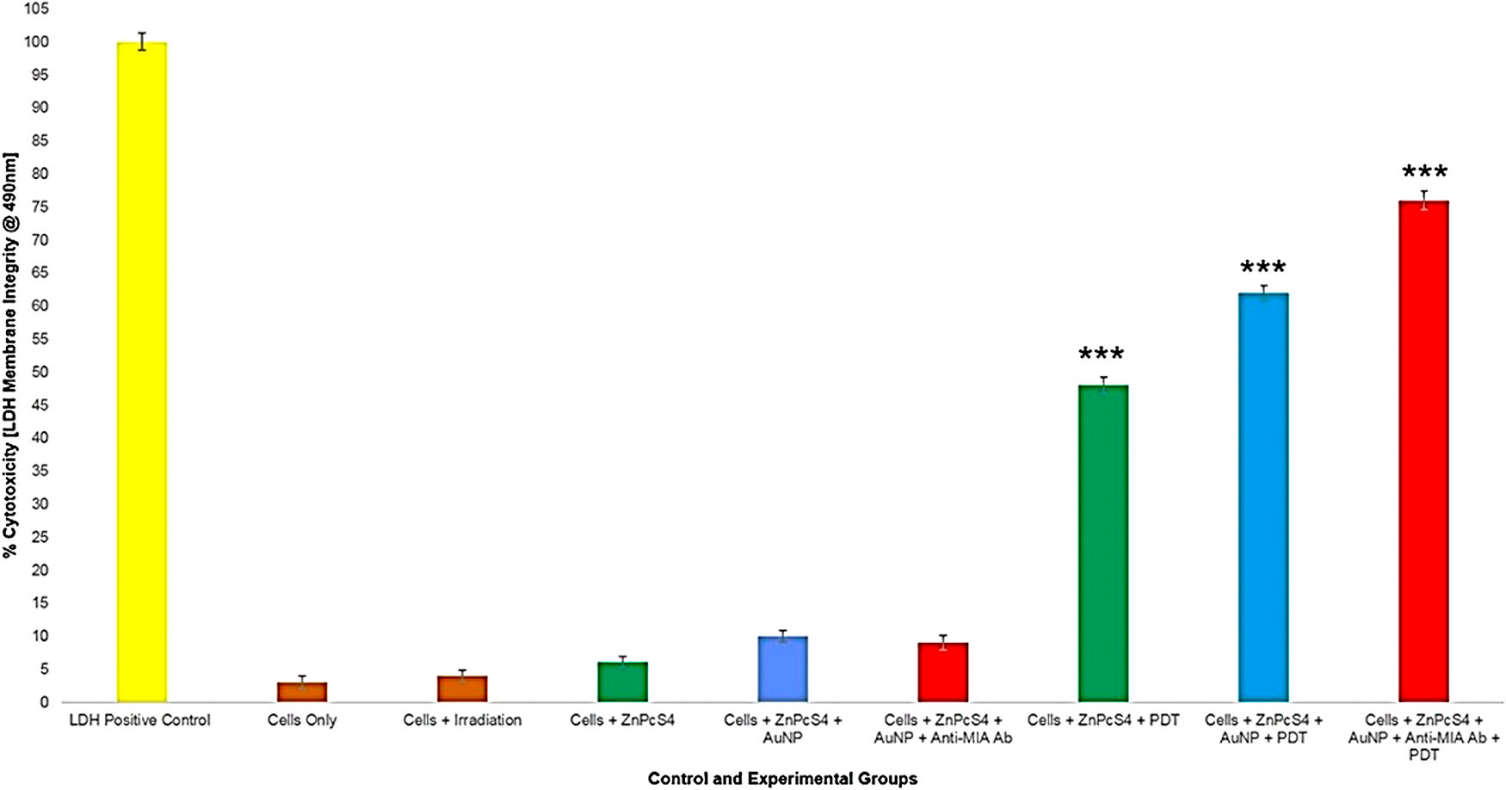
LDH cellular cytotoxicity assay results of control and experimental groups that were subjected to final PS drug conjugate PDT response assays.

In order to determine the cytotoxic effect, the final PS drug conjugate had during PDT response assays, various control and experimental groups were subjected to LDH membrane damage integrity analysis (Figure 13). The MM cells only control group noted negligible cytotoxicity and so this was used as reference-base line to statistically compare other results against. Control groups which received laser irradiation or 2.5 µM ZnPcS_4_ only, no substantial increase in cellular cytotoxicity was found, suggesting that laser treatment alone or PS addition pre-PDT has significant impact on cellular cytotoxicity. Additionally, there was no significant cytotoxicity increase observed in cell control groups, which received control 2.5 µM ZnPcS_4_ – AuNP-PEG5000-SH-NH_2_ or 2.5 µM final PS drug conjugate administration alone. These findings indicate that when the ZnPcS_4_ was bound to either AuNP-PEG5000-SH-NH_2_, and /or Anti-MIA Ab, pre-PDT it remained stable and had no cytotoxic effects on cells.

All PDT treated groups reported highly significant cellular cytotoxicity values which varied over the different groups. Within PDT treated control groups which received 2.5 µM ZnPcS_4_ PS drug only, a significant increase of 45%*** cellular cytotoxicity was noted. However, within PDT treated control groups which received 2.5 µM ZnPcS_4_ – AuNP-PEG5000-SH-NH_2_, a significantly higher increase of 59%*** was found. These findings are supportive of previous results, whereby it has been stated that the binding of AuNPs to PSs stimulate PDT induced photothermal activities, which in turn are cytotoxic to cells [32, 33]. However, the most significant cellular cytotoxicity of 73%*** was noted in PDT treated experimental groups, which received 2.5 µM final PS drug conjugate administration. These findings suggest that the conjugation of Anti-MIA Ab to ZnPcS_4_ – AuNP-PEG5000-SH-NH_2_, actively enhanced PS uptake in MM cells and so improved cellular cytotoxicity post-PDT treatment outcomes significantly.

#### Flow cytometry Annexin V-FITC/PI cell death pathway detection assay

**Figure 14:**
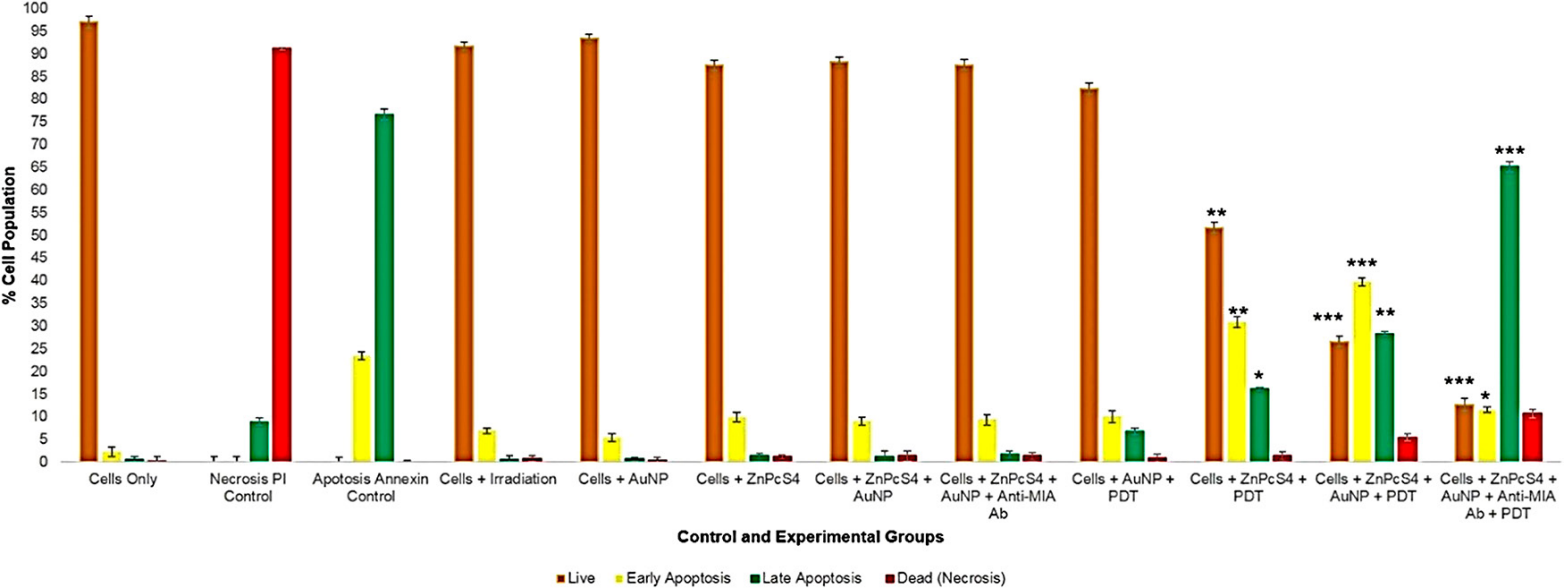
Evaluation of different stages of cell death using flow cytometry Annexin V-FITC/PI staining method on control and experimental groups that were subjected to final PS drug conjugate PDT response assays.

This assay was performed in order determine if the final PS drug conjugate was capable of enhanced PDT induced cell death, due to its functionalized active Ab targeting abilities and AuNP photothermal promoting nanocarrier, when compared to ZnPcS_4_ PS drug or AuNP-PEG5000-SH-NH_2_ administration alone within various control and experimental groups. Flow cytometry scatter grams acquired for the various control and experimental groups were thoroughly analysed, and results have been graphically presented in Figure 14. The control of cells only was used to statistically compare results of viable cells, whereas the necrosis and apoptosis (early and late) controls were used to statistically compare cells undergoing these various modes of cell death. All PDT control and experimental treated groups showed significant modes of cell death when compared to the viable cells only control group, however these significant increases in percentage values varied over the different groups in relation to the mode of cell death each group had undergone.

Within PDT treated control groups which received 2.5 µM ZnPcS_4_ PS drug, only 52%** of the cells were found viable and the remainder were reported in a 32%** early apoptotic and 16%* in a late apoptotic phase of cell death. These results suggest that the administration of ZnPcS_4_ PS drug only with PDT activation has the ability to induce 48% apoptotic cell death in MM cells. These results coincide with studies conducted by Robertson *et al.* (2010) which also reported that PDT laser activated ZnPcS_mix_ administered to melanoma skin cancer cells significantly decreased proportion of viable cells, as well as significantly increased the number of cells undergoing early apoptosis [34]. Nevertheless, studies by Noguchi *et al.* (2015) have stated that cells undergoing early apoptosis modes of cell death, often remain in an autophagy phase and so have the ability to recover [35]. Thus, idealistically, this early mode of apoptotic cell death is not favourable in PDT cancer treatments, as cells can regenerate themselves and so there is a possibility of cancer re-occurrence.

Whereas, within PDT treated control groups which received 2.5 µM ZnPcS_4_ – AuNP-PEG5000-SH-NH_2_, a more significant increase of 27%*** in late apoptosis was noted, with a 40%*** in early apoptosis, 5%** undergoing necrosis and only 28%** of cells being found viable. The improved result of MM cells in late apoptosis when compared to the cells only or PDT treated groups which received 2.5 µM ZnPcS_4_ PS was again attributed to presence of the AuNPs. Studies by El Hussein *et al.* (2015), support these findings stating that within PDT assays performed on cancer cells, that spherical-oval shaped AuNPs demonstrated the ability to activate a PDT photo thermal apoptotic cell death effects, through notable decreases in cell viability and proliferation, as well as an increase in cytotoxicity [36].

However, the most significant modes of cell death was found within PDT treated experimental groups, which received 2.5 µM final PS drug conjugate administration, whereby only 13%*** of the cells were found viable, 12%*** were undergoing early apoptosis and a staggering 64%*** were found in a late apoptotic cell death phase, with a notable 11% increase in necrotic cells. This highly significant result of MM cells undergoing 65% late apoptosis, when compared to the control groups of cells only and PDT treated control groups which received 2.5 µM ZnPcS_4_ PS drug only or 2.5 µM ZnPcS_4_ – AuNP-PEG5000-SH-NH_2_, can be accredited to the fact that ZnPcS_4_ PS drug was conjugated to Anti-MIA Ab and so actively enhanced PS drug uptake. According to studies by Bazak *et al.* (2015) significant progress has been made in terms of specific receptor based PS drug molecular targeting of cancer cells though the conjugation of PSs to nano-carriers, which are further functionalized with Ab biomarkers, to improve PS drug active absorption in targeted cells only, leaving healthy surrounding tissues unaffected and overall improving the late apoptotic cell death mode efficacy induction of PDT tumour treatment [37]. Additionally, studies by Pleshkan *et al.* (2011) indicated that the use of Anti-MIA Ab biomarker conjugated drugs are a promising anti-tumour vehicle for the successful targeted drug delivery and improved treatment of MM [38].

## DISCUSSION

The various molecular characterization assays confirmed that ZnPcS_4_ PS drug was successfully conjugated onto the surface of AuNPs via ligand absorption and exchange methods. Additionally, these same assays noted effective c’ terminus amide bonding of Anti-MIA Abs onto the surface of functionalized amine surface AuNPs, leaving the n’ terminus of the Anti-Ab correctly orientated outwards and unobstructed for active tumor biomarker recognition. DLS and ZP results in relation to the final PS drug conjugate noted that the nanoparticle drug system was homogenously pure, monodispersed and moderately stable and so could be considered as an effective PDT PS drug carrier. Moreover, the subcellular localization results reported that the final PS drug conjugate was more efficient at improved ZnPcS_4_ concentrated cellular absorption within the cytoplasm and nuclei of cells, due to its Anti-MIA Ab biomarker targeting affinity for MM cells.

The findings from this study suggest that the conjugation of Anti-MIA Ab to ZnPcS_4_ – AuNP-PEG5000-SH-NH_2_, within the final PS drug conjugate actively and specifically enhanced ZnPcS_4_ PS drug uptake in MM cells, in comparison to ZnPcS_4_ PS drug administration alone. Thus, the final PS drug conjugate noted significantly enhanced PDT induced cytotoxic cell death in MM cancer cells, in comparison to control groups. These results were also attributed to the PDT induced photothermal cellular destruction abilities of the AuNPs. Additionally, the majority of MM cells which received the final PS drug conjugate and PDT, were found to be undergoing late apoptotic mode of cell death in comparison to ZnPcS_4_ PS drug administration alone. Therefore, the results from this study suggest that the ZnPcS_4_ PS drug delivery within *in vitro* cultured MM cells can be improved using active nanoparticle biomarker targeting and so the overall outcomes of PDT treatment can be enhanced. Additionally, in relation to the findings from this study, the efficacy of MM treatment could possibly be further upgraded in some groups of clinical patients undergoing immunotherapy by utilizing the same Anti-MIA Ab as a molecular target and observing its treatment outcomes [4].

## METHODS

### Chemical synthesis of final conjugate ZnPcS_4_ – AuNP-PEG5000-SH-NH_2_ – Anti-MIA Ab: Conjugation of Anti-MIA Ab to ZnPcS_4_ - AuNP-PEG5000-SH-NH_2_

Working concentration of 0.0005 M ZnPcS_4_ (Santa Cruz®: sc-264509A) (%w/v) in 0.001 M Phosphate Buffer Saline (PBS) was prepared and diluted as needed. 1 ml of AuNP-PEG5000-SH-NH_2_ (Sigma-Aldrich: 765309) which contained 2.85 × 10^15^ AuNPs per ml was added to 1 ml of 0.0005 M stock ZnPcS_4_. It was vortexed at 1 500 rpm at room temperature overnight to promote spontaneous ligand exchange (between Au and PS tetra sulphides) and adsorption (disulphide bond between PEG and PS) (Figure 1). It was purified by micro-centrifugation at 15 200 rpm for 1 hour. The supernatant was discarded and the pellet which contained the conjugated ZnPcS_4_ and AuNP-PEG5000-SH-NH_2_ was re-suspended in 1 ml PBS [26, 39].

200 µg/ml of Anti-MIA Ab (Abcam: 166932) was activated using covalent mode carbodiimide crosslinker two-step coupling EDC and NHS chemistry [23, 25, 40, 41]. The activated c’ terminus succinimidyl ester on the Anti-MIA Ab reacted was then able to react with the amine group (NH_2_) on the AuNPs, already bound in the ZnPcS_4_ – AuNP-PEG5000-SH-NH_2_ conjugate, and so when mixed together, formed a stable amide bond [39]. This method ensured the correct orientation of the bio-targeting antibody, i. e.: the c’ terminus was bound to the amine functionalized AuNPs, while the n’ terminus antigenic sites remained free and functional for active targeting (Figure 1). The final PS drug conjugate (ZnPcS_4_ – AuNP-PEG5000-SH-NH_2_ – Anti-MIA Ab) was then subjected to various molecular characterization assays including; UV-Visible and FT-IR Spectroscopy, DLS and ZP, as well as immunofluorescent staining subcellular localization and uptake confirmation assays.

### Molecular characterization of final conjugate ZnPcS_4_ – AuNP-PEG5000-SH-NH_2_ – Anti-MIA Ab

#### UV-Visible spectroscopy

The UV-Visible absorption and fluorescent spectra of the final conjugate ZnPcS_4_ – AuNP-PEG5000-SH-NH_2_ – Anti-MIA Ab and various controls (ZnPcS_4_, AuNP-PEG5000-SH-NH_2_, Anti-MIA Ab, ZnPcS_4_ - AuNP-PEG5000-SH-NH_2_) were recorded using a Jenway Genova Nano Plus Life Science Spectrophotometer, to observe binding of the Anti-MIA Ab and ZnPcS_4_ PS to the surface of the AuNP-PEG5000-SH-NH_2_. The absorption and fluorescent spectra were read using the spectrum/purity scan mode within the 198 - 800 nm spectral region, as well as read using the 220 nm protein direct UV option. The Anti-MIA Ab protein and ZnPcS_4_ PS concentration, as well as number of bound AuNP to the final conjugate was confirmed, after data interpretation [22]. To determine the photostability of the final conjugate the ZnPcS_4_ – AuNP-PEG5000-SH-NH_2_ – Anti-MIA Ab Soret and Q bands values were measured prior to laser light irradiation experiments over an 8 week period.

#### FT-IR spectroscopy

FT-IR analysis was performed using the Nicolet iS50 FT-IR Spectrometer (Thermo Scientific) to confirm ligand exchange / absorption (by noting and identifying the formation of strong Au-S and weak di-sulphide bonds), as well as confirm amide bond formation (by noting and identifying the formation of amide bonds). The infrared spectra results were recorded using far infra-red solution software at frequencies ranging from 400 - 4000 cm^–1^ with 25 scans.

#### DLS and ZP

DLS and ZP measurements were performed using the Malvern Zetasizer Nano ZS (Malvern Instruments, Malvern UK), which has a 4 mW He-Ne laser of 633 nm wavelength. Samples were heterogeneous or homogenous 10 - 50 µg/ml diluted suspensions in water and were analyzed at 25°C, using a 13° and 173° angel.

#### Subcellular localization

Culture plates were seeded at 2.5 × 10^5^ cells/ml with A375 MM cells, which had sterile coverslips inserted into them and incubated at 37 °C for 4 hours, to allow for cellular attachment. After 4 hours of incubation, the growth media was discarded and replaced with fresh growth media which contained a pre-determined dose response of 2.5 µM PS alone or the final PS drug conjugate to make up a final volume of 3 ml.

The plates were incubated at 37 °C for an additional 20 hours in the dark. After incubation, the primary Ab [200 µg/ml ICAM-1 Mouse Monoclonal IgG1 Ab diluted in 0.01 M PBS in 1% (w/v) BSA and 0.01% (w/v) sodium azide buffer solution, in a ratio 1:200] was added followed by the secondary Ab [200 µg/ml 0.5 ml Goat Anti-Mouse IgG-FITC human absorbed, fluorescein conjugated antibody, diluted in 0.01 M PBS in 1% (w/v) BSA and 0.01% (w/v) sodium azide buffer solution in a ratio 1:200] and then the cells were permanently fixed onto their coverslips. The coverslips were then stained with 50 µl of 1 µg/ml 40-6-Diamidino-2-phenylindole (DAPI) and inverted onto glass microscope slides and sealed with nail varnish. The slides were examined using the filter fluorescent settings of a Carl Zeiss Axio Z1 Observer immuno fluorescent microscope. The 358Ex / 461Em filter was used to detect blue DAPI counter stained nuclei in cultured cells, while the 495Ex / 519Em filter was used to detect any green FITC stained ICAM-1 membrane proteins and the 589Ex / 610Em filter was used to detect any Cy5 red auto fluorescent signal that was produced from the ZnPcS_4_.

### Cell culture and preparation of cell culture plates

Commercially purchased Human malignant melanoma cell line A375 (MM) were obtained from the European Collection of Authenticated Cell Cultures (ECACC no: 88113005) and cultured in a 175 cm^2^ cell culture flask, which contained Dulbecco's Modified Eagle's medium (DMEM) basal medium supplemented with 15% (v/v) Foetal Bovine Serum (FBS), 0.1% (v/v) Amphotericin-β and 0.1% (v/v) Penicillin-Streptomycin. The cultured cells were incubated at 37 °C, with 5% CO_2_ tension and 85% humidity.

Once confluent monolayers of cultured cells were obtained, they were detached using Tryple ™Select. The cellular suspensions were then pelleted out and re-suspended in complete cell growth medium. These cellular suspensions were then sub-cultured into 3.4 cm diameter cell culture plates at a seeding ratio of 2.5 × 10^5^ cells/ml, which contained 3 ml of complete cell growth medium. The culture plates were then incubated for 4 hours to allow for cellular attachment.

This particular cell line was purchased from ECACC in March 2018 and was passaged within these laboratory experiments for fewer than 6 months after receipt and so re-authentication was not required. However, the ECACC cell bank notes that they utilize human cell lines Short tandem repeat (STR) loci human cell line profiling methods of characterization to ensure cell line authenticity.

### PDT laser parameters, PS drug addition to culture plates and laser irradiation

After 4 hours incubation, culture plates were divided into various control and experimental groups, for ZnPcS_4_ PDT PS dose response or final PDT PS drug conjugate assays. Then the cell culture media was freshly replaced. The groups which required PS or final PS drug conjugate, had the required concentration added to their culture media and were incubated for an additional 20 hours. Then the groups which required laser treatment (wavelength: 673 nm and fluency:10 J/cm^2^) were irradiated for approximately 16 minutes and 8 seconds in 1 ml 0.001 M PBS using a Roithner 1000 mA 673 nm high power semiconductor diode laser (Arroyo 4210). The culture media of all plates was then freshly replaced, and plates were incubated for an additional 24 hours.

#### ZnPcS_4_ PS PDT dose response assays

To determine the IC_50_ of PS PDT treatment alone a concentration range between 0.25 – 10 µM of ZnPcS_4_ was administered to various control and experimental culture plate groups, as discussed above. Post laser irradiation (for some groups) and after an additional 24 hours of incubation, following detachment and cellular suspension these groups were subjected to Trypan blue and cytotoxicity analysis. Note: the IC_50_ of ZnPcS_4_ was found to be 2.5 µM.

#### ZnPcS_4_ – AuNP-PEG5000-SH-NH_2_ – Anti-MIA Ab final PS drug conjugate PDT response assays

Thus, to determine if the final PS drug conjugate enhanced PDT treatment in comparison to PS PDT treatment alone, 2.5 µM of the final PS drug conjugate and/or laser light irradiation was administered to various control and experimental culture plate groups, as discussed above. Post laser irradiation (for some groups) and after an additional 24 hours of incubation, following detachment and cellular suspension these groups were subjected to various biochemical assays.

### Biochemical assays

#### Morphology

Post-PDT treatment, the effect on cellular morphology was viewed by light microscopy and observed at 400× magnification using an inverted microscope (Olympus CKX41) that had a digital camera (Olympus C5060-ADUS) attached to it.

#### The trypan blue exclusion test

10 ul of 0.4% (w/v) Trypan Blue stain was mixed with 10 µl of cellular suspensions and loaded onto a disposable Countess® cell counting chamber slide. The number of viable cells per ml was quantified using an Invitrogen Countess® II FL Automated Cell Counter.

#### LDH cellular cytotoxicity and membrane integrity assay

The CytoTox96® Non-Radioactive Cytotoxicity Assay (Promega, G1780), measures the amount of lactate dehydrogenase (LDH) that is released into culture medium upon cell lysis and so can be used to determine cellular cytotoxicity. Thus, following manufacturer’s instructions, 50 µl of complete cell culture media from each experimental and control culture plate was removed and added to 50 µl of LDH Reconstituted Substrate Mix in a flat 96 well clear bottom plate. LDH absorbance was recorded at 490 nm using a spectrophotometer (Perkin Elmer, Victor^3^, 1420 Multilabel Counter) and so used to calculate cellular lysis and cytotoxicity.

#### Flow cytometry Annexin V-FITC/PI cell death pathway detection assay

Following manufacturer’s instructions, the Annexin V-FITC/PI cell death detection kit (BD Scientific: BD/556570), was used to detect early or late apoptotic, as well as necrotic phases of cells death in various control and experimental groups, using the BD Accuri^™^ C6 flow cytometer.

### Statistical analysis

Graphs represent the mean and the standard deviation of biochemical assays done in duplicate for six independent experiments. The Students t-test and one-way analysis of variances (ANOVA), was used for normal distributed data, whereas the Mann-Whitney test was used for non-normal distributed data. These tests were used to determine the significance difference between control and experimental groups where values in the 95% confidence interval (P < 0.05*, P < 0.01** or P < 0.001***) were accepted as statistically different.

## Abbreviations

MM: Metastatic melanoma
PDT: Photodynamic Therapy
PS: Photosensitizer
nm: nanometre
ROS: Reactive Oxygen Species
ZnPcS /ZnPcS_mix_: Zinc sulpho phthalocyanine
AuNP: Gold nanoparticle
MIA: Melanoma Inhibitory Activity
Ab: Antibody
Anti-MIA: Anti-Melanoma Inhibitory Activity
ZnPcS_4_: Zinc phthalocyanine tetra-sulphonic acid
AuNP-PEG5000-SH-NH_2_: Amine functionalized pegylated gold nanoparticle
UV: Ultra Violet
µM: Micromolar
PEG: Polyethylene glycol
C: Carbon
S: Sulphur
H: Hydrogen
N: Nitrogen
NH_2_: Anime
ml: millilitre
µg: Microgram
O: Oxygen
n’: terminus Amino terminus
c’: terminus Carboxyl terminus
FT-IR: Fourier Transform Infrared
Au: Gold
DLS: Dynamic Light Scattering
ZP: Zeta Potential
PDI: Polydispersity Index
mV: millivolt
IC_50_: Inhibitory Concentration of 50%
J/cm^2^: Joules per cm^2^
LDH: Lactate Dehydrogenase
FTIC: Fluorescein isothiocyanate
PI: Propidium iodide
M: Molar
PBS: Phosphate Buffered Saline
rpm: rotation per minute
mg: milligrams
EDC: 1-Ethyl-3-(3-dimethylaminopropyl)carbodiimide
NHS: N-Hydroxysuccinimide
µl: Microliter
°C: Degree Celsius
mW: milliwatt
He-Ne: Helium-neon
ICAM-1: Intercellular adhesion molecule-1
BSA: Bovine serum albumin
DAPI: 4',6-diamidino-2-phenylindole
ECACC: European Collection of Authenticated Cell Cultures
DMEM: Dulbecco's Modified Eagle's medium
FBS: Foetal Bovine Serum
CO_2_: Carbon dioxide
mA: milliampere
BD: Becton Dickinson
ANOVA: Analysis of variances
